# A Case of Granulicatella adiacens Osteomyelitis and Mitral Valve Endocarditis

**DOI:** 10.7759/cureus.68331

**Published:** 2024-08-31

**Authors:** Michelle Baek, Dima Youssef

**Affiliations:** 1 Family Medicine, Valley Health System, Las Vegas, USA; 2 Internal Medicine, Valley Health System, Las Vegas, USA

**Keywords:** mitral valve endocarditis, discitis, lumbar spine discitis, bacteremia, endocarditis, osteomyelitis, granulicatella adiacens

## Abstract

*Granulicatella adiacens* is a nutritionally variant streptococci (NVS) that can cause various infections, including but not limited to endocarditis, osteomyelitis, pneumonia, and abscess. We report a case of an 80-year-old male who was found to have *Granulicatella adiacens* osteomyelitis and mitral valve endocarditis. Also included is a systematic review of osteomyelitis caused by *Granulicatella adiacens.*

## Introduction

*Granulicatella adiacens* can cause various infections such as osteomyelitis, endocarditis, abscesses, pneumonia, and more [1-3]. Concurrent infections, including multiple systems, are possible and have been reported. Depending on the type of infection, most cases can be treated successfully with intravenous antibiotics [2]. However, there are cases necessitating surgical interventions for full recovery. Here, we present a case of an 80-year-old male with a history of hypertension and transcatheter aortic valve replacement (TAVR) who was found to have *Granulicatella adiacens* bacteremia causing lumbar spine osteomyelitis/discitis and mitral valve endocarditis.

## Case presentation

This is an 80-year-old male with a significant cardiac history (TAVR within the last 10 years and controlled hypertension) who presented to the hospital with altered mental status and subjective fevers. A few weeks prior to admission, the patient stated he had an MRI of his lumbar spine completed due to new onset low back pain. According to the patient, the MRI showed no acute findings. Prior to this, the patient had been living an active lifestyle - engaging in daily walks and attending Pilates classes. On admission, labs and vitals were remarkable for fever of 100.9°F, heart rate of approximately 120 bpm, and elevated troponin levels. Table 1 shows the lab results during admission. Throughout the hospital stay, he did not have leukocytosis. Sepsis workup, including a COVID-19/flu test, urinalysis, and chest X-ray, did not show any signs of infection. Two blood cultures drawn on admission grew *Granulicatella adiacens*. The patient was admitted for sepsis workup as well as cardiac evaluation and was found to have a rare bacteremia secondary to discitis.

**Table 1:** Laboratory results from admission UA - Urinalysis, Ur - Urine

Parameter	Result	Parameter	Result
White blood cells	12.40	Ur color	Yellow
Red blood cells	3.46	Ur clarity	Clear
Hemoglobin	10.0	Ur glucose	Negative
Hematocrit	30.2	Ur bilirubin	Negative
Platelet	160	Ur ketones	Negative
Troponin	276.8	Ur specific gravity	1.021
Troponin	1,406.4	Ur blood	Negative
Troponin	1,059.2	Ur pH	5.0
Glucose	117	Ur protein	1+
Sodium	139	Ur urobilinogen	<2
Potassium	3.7	Ur nitrite	Negative
Chloride	109	Ur leukocyte esterase	Negative
HCO_3_^-^	25	UA white blood cells	2
Anion gap	5	UA red blood cells	<1
Blood urea nitrogen (BUN)	23	Ur bacteria	None seen
Creatinine	1.160	Ur squamous epithelial cells	None seen
BUN/creatinine ratio	20	Ur mucous	Rare
Calcium	8.2	Ur hyaline cast	3

**Figure 1:**
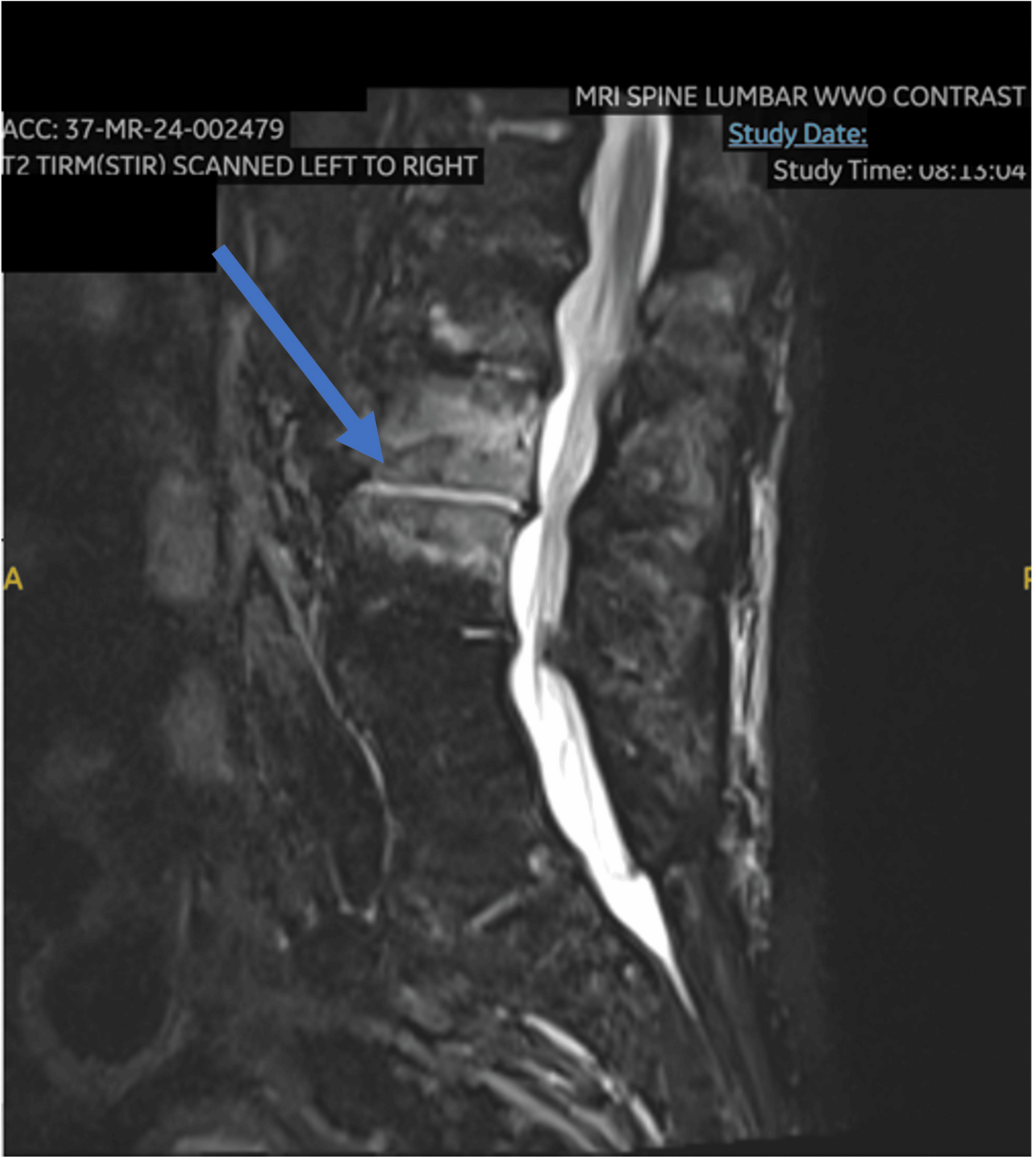
MRI of lumbar spine showing L2-3 osteomyelitis/discitis

**Figure 2:**
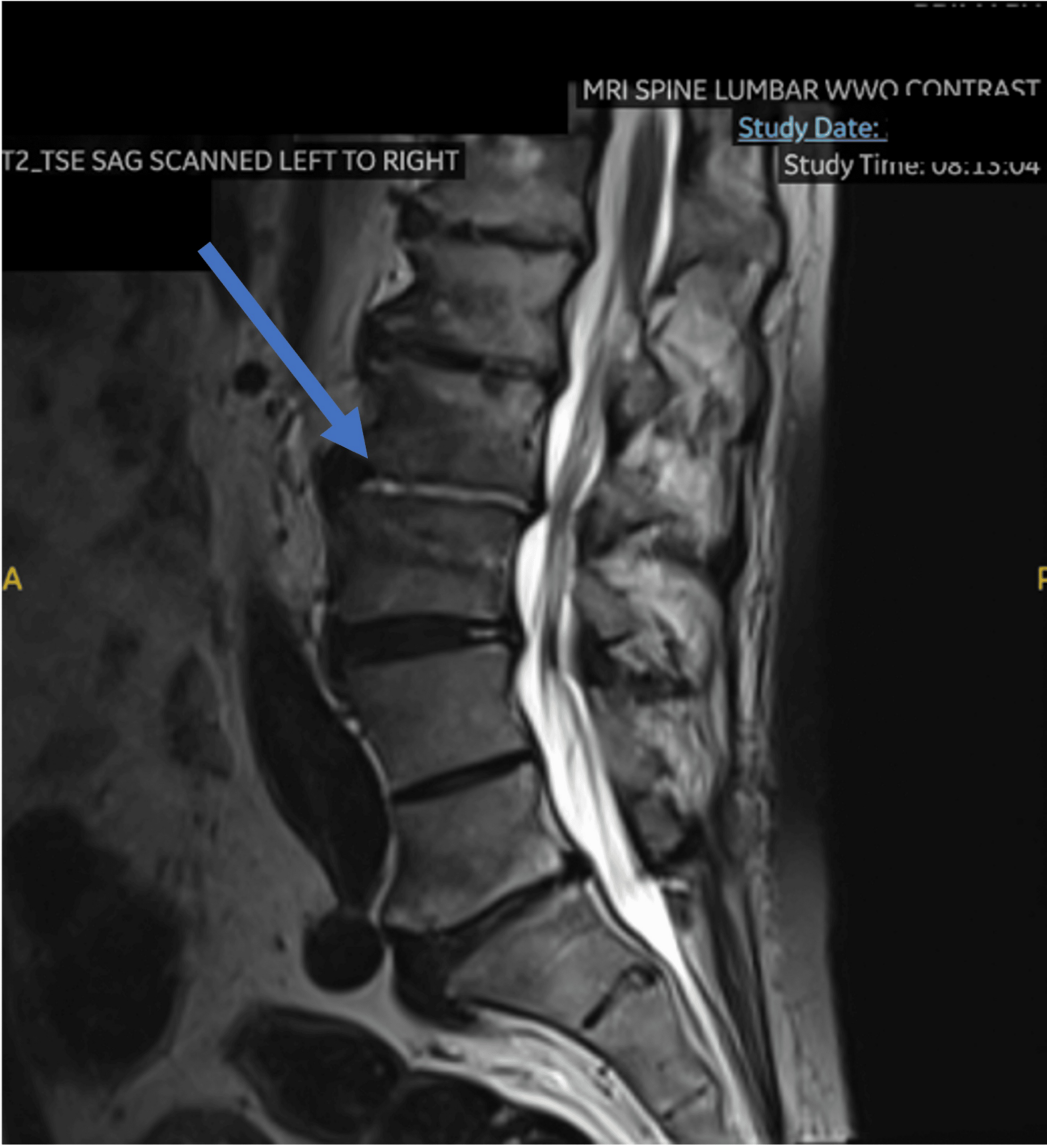
MRI of lumbar spine showing L2-3 osteomyelitis/discitis

**Figure 3:**
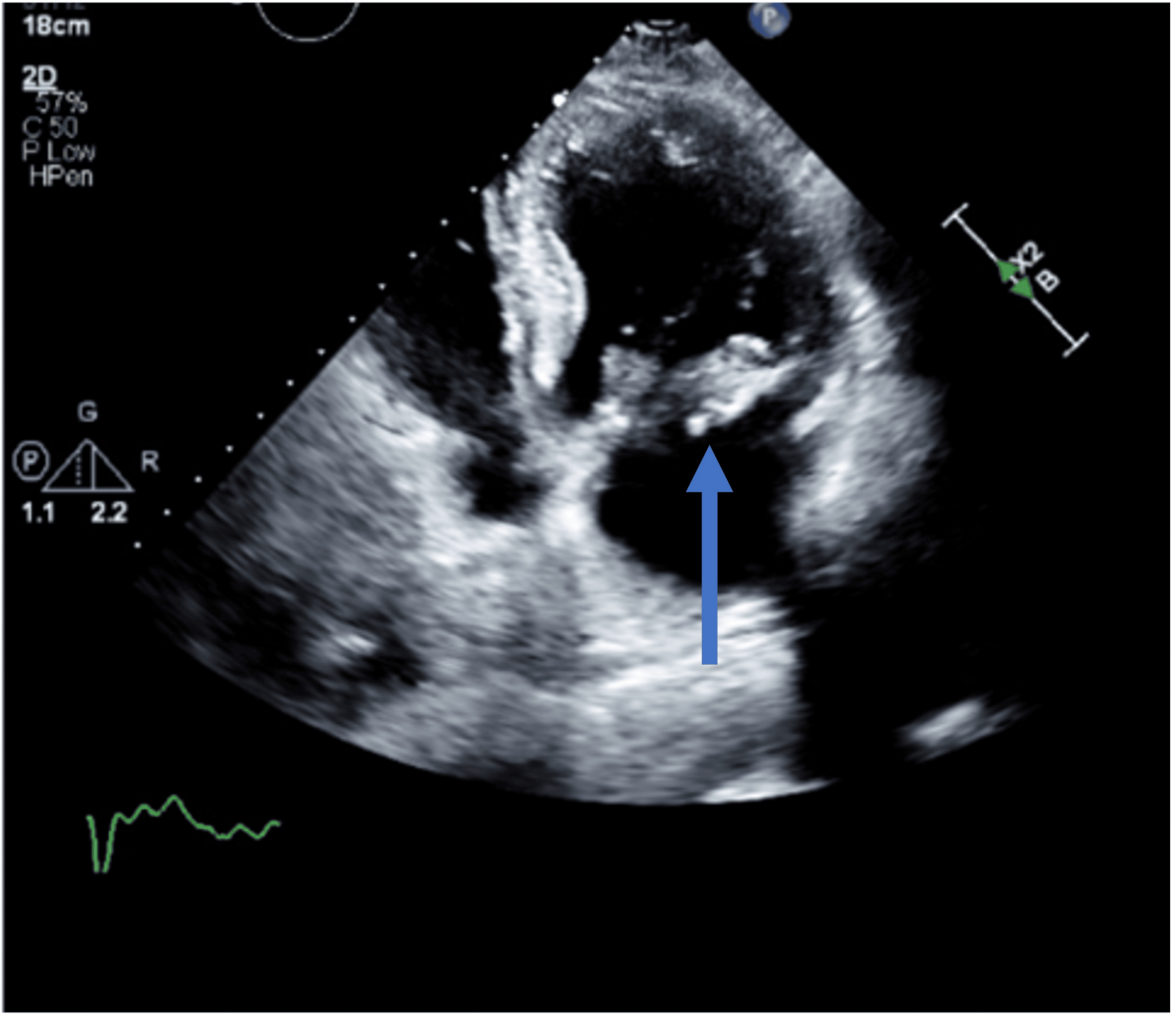
Echocardiogram of mitral valve vegetation

Due to his acute onset low back pain, an MRI of his lumbar spine was obtained, which showed signs of osteomyelitis/discitis on the L2-L3 level (Figure 1 and Figure 2). A cardiac workup was also pursued. A heparin drip was started for the elevated troponins while awaiting an official cardiology consultation. A transthoracic echocardiogram was ordered, which showed preserved ejection fraction, mild concentric left ventricular hypertrophy, moderate to severe mitral regurgitation, TAVR, and medium-sized vegetation on the mitral valve (Figure 3). Per the cardiologist, the elevation in troponin levels was likely due to non-ST-elevation myocardial infarction (NSTEMI) type II strain. Cardiovascular thoracic surgery consultation was offered, but the patient elected for non-surgical management, choosing to follow up with his established cardiologist once outpatient. Eventually, the patient was discharged with a six-week course of vancomycin via a peripherally inserted central catheter (PICC) line. Following the treatment, he made a full recovery. His symptoms improved significantly, and he was back to his baseline in terms of activities.

## Discussion

*Granulicatella adiacens*, a nutritionally variant streptococci (NVS) and part of the normal flora of the oral cavity and intestinal tract, can cause various infections including but not limited to endocarditis, osteomyelitis, pneumonia, and abscess [1-3]. A singular infection may develop; however, concurrent infections of multiple systems are possible, especially in immunocompromised individuals [1,2]. A systematic review of literature in PubMed, National Institutes of Health (NIH), and Google Scholar databases for osteomyelitis caused by *Granulicatella adiacens* yielded 10 articles, 11 cases (two separate cases in one report) [4-13]. Our case marks the 12th case. Three out of 11 published cases reported concurrent endocarditis [4,10,11]. Most of the antibiotic treatment duration was six weeks, with some additions. The preferred antibiotics for treatment are vancomycin (six out of 11), gentamicin (four out of 11), and penicillin (three out of 11). It is also important to note the usage of cephalosporin in five out of 11 cases. An article published by Mushtaq et al. in 2016 reported susceptibilities of *Granulicatella adiacens* to be 34% to penicillin, 22% to ceftriaxone, 3% to cefepime, 87% to meropenem, 10% to cefotaxime, 97% to levofloxacin, 80% to clindamycin, and 50% to erythromycin. All *Granulicatella adiacens* isolates were susceptible to vancomycin [14]. Table 2 outlines the details of the treatments, including sequence and duration.

**Table 2:** Reported cases of Grannulicatella adiacens osteomyelitis AMOX - Amoxicillin, AMP - Ampicillin, CAZ - Ceftazidime, CLINDA - Clindamycin, CPM - Cefepime, CTX - Ceftriaxone, ETP - Ertapenem, GENT - Gentamicin, LEV - Levofloxacin, MERO - Meropenem, MVR - Mitral valve replacement, PCN - Penicillin, RIF - Rifampin, TEC - Teicoplanin, VANC - Vancomycin

Author	Gender	Age	Endocarditis	Bacteremia	Levels involved	Antibiotic tx	Duration
Rosenthal O et al. [4], 2002	M	68	Yes	Yes	T10-11	PCN + IV GENT + RIF	Unknown
Fukuda R et al. [5], 2010	M	73	No	Yes	L3-L4	PCN G + IV GENTfollowed by PO AMOX^a^	6 weeks: a - 9 weeks
York J et al. [6], 2016	M	46	No	No	L2	IV VANC +CAZ^a^	6 weeks: a - only pre-operatively
Bakhsh et al. [7], 2017	M	48	No	No	L3-L5	IV VANC + CPM	6 weeks
Sandhu R. et al. [8], 2017	M	61	No	No	L3-L4	IV CTX + GENT	6 weeks
Perna et al. [9], 2020	M	51	No	Yes	L1-L2, L5-S1	IV TEC^a^ + PO LEVO^b^	6 weeks: a - 1000 mg q12h x 3 doses, then 1000 mg q24h; b - 750 mg q24hrs
Patil et al. [10], 2019	F	44	Yes	Yes	L3-L4	IV VANC^a^ IV MERO^b^ IV VANC + ETP^c^	6 weeks: a - 1.5g q12h starting day 5; b - 2g q8h starting day 9; c - s/p MVR
Puzzolante et al. [11], 2019	M	50	Yes	Yes	L3-L4, L5-S1	IV VANC^a^ + IV CTX^b^+ GENT^c^ followed by IV AMP^d^ followed by PO AMOX^e^	6 weeks: a - 2g qd; b - 2g qd; c - 5 mg/kg/qd × 2 weeks; d - 12 g/qd × 2 weeks; e - 4g/qd × 2 weeks
Puzzolante et al. [11], 2019	M	47	No	Yes	L5-S1	VANC^a^+ CTX^b^ followed by IV CTX^c^ followed by PO AMOX^d^	6 weeks: a - 2g qd × 1 week; b - 2g qd × 1 week; c - 3 weeks; d - 3g qd × 2 weeks
Palen et al. [12], 2021	M	45	No	Yes	L2-L3	PCN + IV GENT^a^ followed by IV PCN^b^ followed by PO CLINDA^c^	5 weeks: a - GENT 3 mg/kg/24 hours on admission; b - 3 weeks; c - 600 mg q8h × 2 weeks
Kuo et al. [13], 2020	F	40	No	Yes	L5-S1	IV VANC followed by PO AMOX^a^	Unknown: a - 14 days unknown

## Conclusions

*Granulicatella adiacens* is a gram-positive bacterium, also known as NVS, that is a cause of infectious conditions such as endocarditis, osteomyelitis, abscess, etc. We learned through our systematic review that most cases are treated with antibiotics with or without concurrent surgical interventions. It is important to note that although the use of decompressive surgery, laminectomy, or discectomy is common, patients may be able to achieve complete recovery with the use of IV antibiotics alone. This can be considered in patients who are considered at high risk for operative complications.
